# Induced tolerance to abiotic and biotic stresses of broccoli and *Arabidopsis* after treatment with elicitor molecules

**DOI:** 10.1038/s41598-020-67074-7

**Published:** 2020-06-25

**Authors:** Jhon Venegas-Molina, Silvia Proietti, Jacob Pollier, Wilson Orozco-Freire, Darío Ramirez-Villacis, Antonio Leon-Reyes

**Affiliations:** 1 Laboratorio de Biotecnología Agrícola y de Alimentos-Ingeniería en Agronomía, Colegio de Ciencias e Ingenierías El Politécnico, Universidad San Francisco de Quito USFQ, Campus Cumbayá, 17-1200-841 Quito, Ecuador; 2 Plant-Microbe Interactions, Department of Biology, Science4Life, Utrecht University, Utrecht, The Netherlands; 3 Ghent University, Department of Plant Biotechnology and Bioinformatics, 9052 Ghent, Belgium; 4 VIB Metabolomics Core, 9052 Ghent, Belgium; 5 Present Address: Department of Ecological and Biological Sciences, University of Tuscia, Viterbo, Italy

**Keywords:** Physiology, Plant sciences, Environmental sciences

## Abstract

The plant hormones salicylic acid (SA) and jasmonic acid (JA) regulate defense mechanisms capable of overcoming different plant stress conditions and constitute distinct but interconnected signaling pathways. Interestingly, several other molecules are reported to trigger stress-specific defense responses to biotic and abiotic stresses. In this study, we investigated the effect of 14 elicitors against diverse but pivotal types of abiotic (drought) and biotic (the chewing insect *Ascia monuste*, the hemibiotrophic bacterium *Pseudomonas syringae* DC 3000 and the necrotrophic fungus *Alternaria alternata*) stresses on broccoli and *Arabidopsis*. Among the main findings, broccoli pre-treated with SA and chitosan showed the highest drought stress recovery in a dose-dependent manner. Several molecules led to increased drought tolerance over a period of three weeks. The enhanced drought tolerance after triggering the SA pathway was associated with stomata control. Moreover, methyl jasmonate (MeJA) reduced *A. monuste* insect development and plant damage, but unexpectedly, other elicitors increased both parameters. GUS reporter assays indicated expression of the SA-dependent *PR1* gene in plants treated with nine elicitors, whereas the JA-dependent *LOX2* gene was only expressed upon MeJA treatment. Overall, elicitors capable of tackling drought and biotrophic pathogens mainly triggered the SA pathway, but adversely also induced systemic susceptibility to chewing insects. These findings provide directions for potential future in-depth characterization and utilization of elicitors and induced resistance in plant protection.

## Introduction

Plant development is extensively affected by abiotic stresses such as heat, flooding, drought, salinity, and a broad spectrum of plant pathogens and herbivorous insects^1,2^. In agriculture, the net production of major crops is significantly reduced by these unfavorable factors^3^. However, over the centuries, plants have evolved and developed inducible defense mechanisms to tackle pathogen infections, herbivore attack, and environmental constraints^4^. These defensive mechanisms are regulated by complex signaling pathways, molecules, and transcriptional regulators^5,6^. Specifically, the plant hormones salicylic acid (SA), jasmonic acid (JA) and ethylene (ET) are described as the central regulators of induced defense responses^5,6^. For instance, upon a biotrophic infection, the first local defense barrier is a hypersensitive response (HR), which regulates cell death at the infection site^7^. Subsequently, the accumulation of SA initiates the secondary defense system, triggering the chemical defense arsenal and reducing further infections in distal tissues^8^. Hence, plants can change from a passive to an active defense state as a result of the initial perception of a given stimulus and its subsequent signaling transduction^6^.

One of the best characterized induced defense systems is called systemic acquired resistance (SAR), which is triggered by the perception of biotrophic pathogens and is mediated by a signaling process controlled by the SA phytohormone^7^. The SAR defense response is mainly effective against biotrophic and hemibiotrophic pathogens, including bacteria^9^, viruses^10^, fungi^11,12^, and phloem-feeding insects^13^. An intact structure of the phloem is required for the activation of SAR since it is the path for communication between the tissues infected by the pathogen and the uninfected distal tissues. Molecules such as pipecolic acid play essential roles in the translocation of the long-distance signals via the phloem and the amplification of the immunity signal^14^. In addition to SAR, a second induced resistance system known as induced systemic resistance (ISR) can be activated by non-pathogenic rhizobacteria. ISR is mainly effective against necrotrophic pathogens and chewing insects, and its response is mediated by the JA and ET hormones^15,16^. The ISR is characterized by the induction of a faster and more effective response against biotic stress as the plant cells are activated prior to the stress by non-pathogenic rhizobacteria. This concept is called priming^17^ and was initially discovered in studies on the interplay of plants with beneficial soil microorganisms such as *Pseudomonas fluorescence* or *Trichoderma* sp^18^.

In addition to pathogens and non-pathogenic agents, the SAR an ISR plant defense pathways can also be triggered by elicitor molecules^19^. The use of elicitor molecules in agriculture may offer advantages such as resistance against a plethora of threats at the same time (microbes, insects, nematodes, environmental stress), long-lasting effects, relatively low prices, and ecological compatibility acceptable for the organic industry^20,21^. Unfortunately, the use of elicitor molecules in agriculture is restricted owing to the lack of basic knowledge on the responses to different kinds of stress, optimal concentrations, number of applications, and effectiveness in different environmental conditions^21^. Some studies suggest that the disease control achieved by the exploitation of induced resistance may range from 20 to 85%, depending on the environmental conditions and genotype^2,20,21^, but the evidence is not clear. To maximize the potential of elicitor molecules, it is vital to understand their effects on plant-pathogen and plant-insect interactions and several environmental stresses. The use of elicitor molecules may lead to broad-spectrum and long-lasting natural plant protection and may ultimately lead to a reduced application of chemical pesticides^19^. Hence, to be able to exploit the potential benefits of elicitor molecules to stimulate plant defense, an extensive characterization of the elicitor molecules against various plant biotic attackers and upon several environmental conditions, is needed.

In this study, we characterized the effectiveness of 14 elicitor molecules against a broad spectrum of biotic and abiotic stresses using the commercial crop broccoli (*Brassica oleracea* var. *italica*) and the model plant *Arabidopsis thaliana* under similar experimental conditions. The effect of 14 elicitor molecules was assessed on different types of stress encountered by plants, including drought, infestation with the chewing insect *Ascia monuste*, infection with the hemibiotrophic bacterium *Pseudomonas syringae* pv. *tomato* DC3000 and the necrotrophic fungus *Alternaria alternata*. Furthermore, *Arabidopsis* reporter lines with GUS fused to the promoters of defense marker genes were evaluated to identify the induced metabolic defense pathway. Our results reveal crucial effects of the use of elicitors on plants challenged with different stresses, sometimes leading to increased plant resistance, but in some cases, also leading to increased susceptibility of the treated plants. Furthermore, our data evidence that the induced drought tolerance is at least in part regulated via the SA pathway and that it is associated with stomata control.

## Results

### Drought stress tolerance in broccoli after elicitor treatment

Based on a literature survey, 14 elicitor molecules that were shown to enhance stress tolerance by activation of the plant defense systems were selected:

1. Acibenzolar S-methyl (ASM), a fungicide that acts by activation of SAR^22^.
2. *β*-Aminobutyric acid (BABA), a non-protein amino acid that induces systemic resistance^23,24^.
3. Saccharin (Sacch), a well-known artificial sweetener that induces systemic resistance^25^.
4. Riboflavin (Rib) or vitamin B_2_, which induces priming of defense responses independent of JA or SA^26^.
5. Hexanoic acid (Hx), a natural carboxylic acid that induces pathogen resistance in plants by enhancing JA-dependent defenses^27^.
6. Sodium silicate (Si), as silicon, was shown to increase ISR in plants^28,29^.
7. Menadione sodium bisulfite (MSB), a water-soluble addition compound of vitamin K_3_ that induces resistance to fungi and a priming agent for improving salt stress tolerance^30,31^.
8. Chitosan (CHT), a natural linear polysaccharide that induces biotic and abiotic stress tolerance^32,33^.
9. Azelaic acid (AzA), a nine-carbon dicarboxylic acid that serves as a mobile SAR signal in the vascular sap and that confers local and systemic resistance to biotic stress^34,35^.
10. Potassium phosphite (KP), a phosphite salt used as an antifungal agent, and induced SA-mediated defense^24,36^.
11. Calcium phosphite (CaP), a phosphite salt that, like KP, induces resistance against fungi^36^.
12. Thiamine (Thiam) or vitamin B1 that functions as an activator of plant disease resistance^37^.
13. Salicylic acid (SA), a plant hormone that plays a central role in plant defense responses against biotrophic pathogens^38^.
14. Methyl jasmonate (MeJA), a derivative of jasmonic acid implicated in plant defense against necrotrophic pathogens and insects^38^.

**Figure 1:**
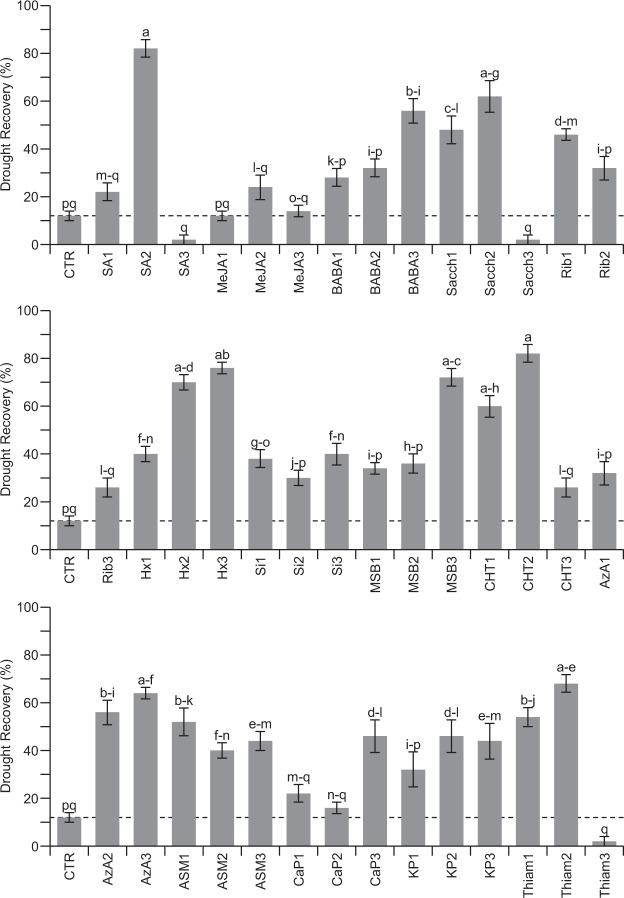
Evaluation of drought stress tolerance in broccoli after elicitor treatment. Three different concentrations per elicitor molecule were tested (Table 1). The dashed line indicates the mock control level. The error bars designate the standard error (n = 5 biological replicates of 10 plants each). The different letters indicate the statistical significance determined by ANOVA (*P* < 0.001) with a post-hoc Tukey test (*P* < 0.05). All three panels belong to the same experiment, but the data were divided for a better graphic representation. The control is the same for all of the treatments, and letters represent the statistical significance for the entire experiment.

To assess the effect of the selected elicitor molecules on the recovery of broccoli from drought stress, 14-day-old broccoli plants were pre-treated with the different elicitors at different concentrations (Table 1), after which water was withheld until the mock-treated plants reached a relative water content (RWC) of 30%, that in our setup corresponded to 7 to 8 days of water deprivation. After this drought period, the broccoli plants were re-watered, and 24 hours after watering, their recovery from the drought period was registered (Fig. 1). Compared to mock-treated control plants, most of the broccoli plants that were pre-treated with the selected compounds showed enhanced recovery from the applied drought stress (Fig. 1). More specifically, from the 42 different treatments (Table 1), 23 led to a significantly enhanced drought stress recovery (Fig. 1). Besides, most of the treatments that were not significantly different from the control also showed a trend towards an enhanced drought tolerance. The type of elicitor and its concentration directly affected the recovery effect, suggesting that dose is a critical factor for the induction of drought tolerance. For instance, in the SA2 and CHT2 treatments, plants showed the highest drought stress tolerance with 82% recovery, compared with the control treatment that led to only 12% recovery (Fig. 1). Conversely, only three treatments: SA3, Sacch3, and Thiam3, displayed visual leaf damage, possibly due to toxic effects of high concentrations of the applied elicitors.

**Table 1:** Elicitor molecules and applied concentrations in the drought stress screening experiments.

Elicitor	Concentration 1	Concentration 2	Concentration 3	References
SA	0.3 mM	1.0 mM^a^	3.0 mM	^83^
MeJA	0.03 mM	0.1 mM^a^	0.3 mM	^83^
BABA	0.1 mM	0.3 mM	1.0 mM^a^	^23^
Sacch	5.4 mM	16.3 mM^a^	54.6 mM	^25^
Rib	0.1 mM^a^	0.3 mM	1.0 mM	^26^
Hx	0.6 mM	2.0 mM	6.0 mM^a^	^27^
Si	16.7 mM	50.0 mM	166.7 mM^a^	^28^
MSB	0.06 mM	0.2 mM	0.6 mM^a^	^30^
CHT	0.3 mM	0.9 mM^a^	2.7 mM	^32^
AzA	0.3 mM	1.0 mM	3.0 mM^a^	^34^
ASM	0.04 mM^a^	0.14 mM	0.5 mM	^22^
CaP	1.3 mM	4.3 mM	12.9 mM^a^	^36^
KP	3.9 mM	13.0 mM^a^	39.0 mM	^24^
Thiam	16.6 mM	50.0 mM^a^	150.0 mM	^37^

^a^Concentration leading to the best drought stress tolerance that was used for all the following experiments.

### Persistence of induced drought stress tolerance in broccoli

**Figure 2:**
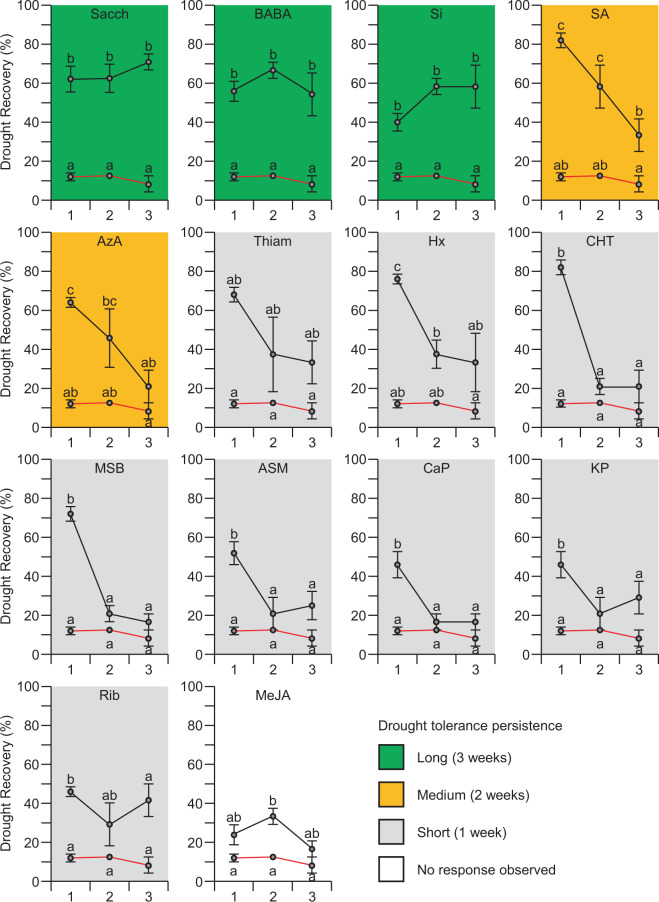
Persistence of induced drought stress tolerance in broccoli up to three weeks after elicitor treatment. Recovery from drought stress applied one, two, and three weeks after elicitor treatment was scored. The molecules were classified according to the observed recovery pattern. The red graph in each plot represents the mock treatment. The error bars designate the standard error (n = 5 biological replicates of 10 plants each). For clarity, we included in the figure legend: All panels belong to the same experiment, but the data were divided for a better graphic representation. In addition, statistical analysis was performed using ANOVA (*P* < 0.001) with a post-hoc Tukey test (*P* < 0.05) comparing each elicitor treatment and mock independently.

To evaluate the persistence of drought stress tolerance over time, plants were subjected to drought stress one, two, or three weeks after mock or elicitor treatment. For this experiment, the selected elicitor concentration was the concentration with the highest recovery response from the previous drought experiment (Fig. 1; Table 1). Like in the last experiment, drought stress was applied by withholding water until the mock-treated plants reached a relative water content (RWC) of 30%. Twenty-four hours after re-watering, the recovery from the applied drought stress was evaluated (Fig. 2). The drought tolerance of the broccoli plants was reduced in all treatments over time, however, for certain elicitor molecules, enhanced drought tolerance was observed up to three weeks after the elicitor application. Overall, four distinct recovery patterns could be observed (Fig. 2):

1. Long-term drought tolerance: increased drought tolerance was observed up to three weeks after treatment with BABA, Sacch, or Si.
2. Medium-term drought tolerance: enhanced drought tolerance the first two weeks, but sensitive to drought stress in the third week after treatment with SA or AzA.
3. Short-term drought tolerance: enhanced drought tolerance only in the first week after treatment with Thiam, Hx, CHT, MSB, ASM, CaP, KP, or Rib.
4. No improved drought tolerance: MeJA.

Together, these results show that besides the concentration, also the type of elicitor molecule is critical for induced drought tolerance over time.

### Resistance to chewing insects after elicitor treatment in broccoli

**Figure 3:**
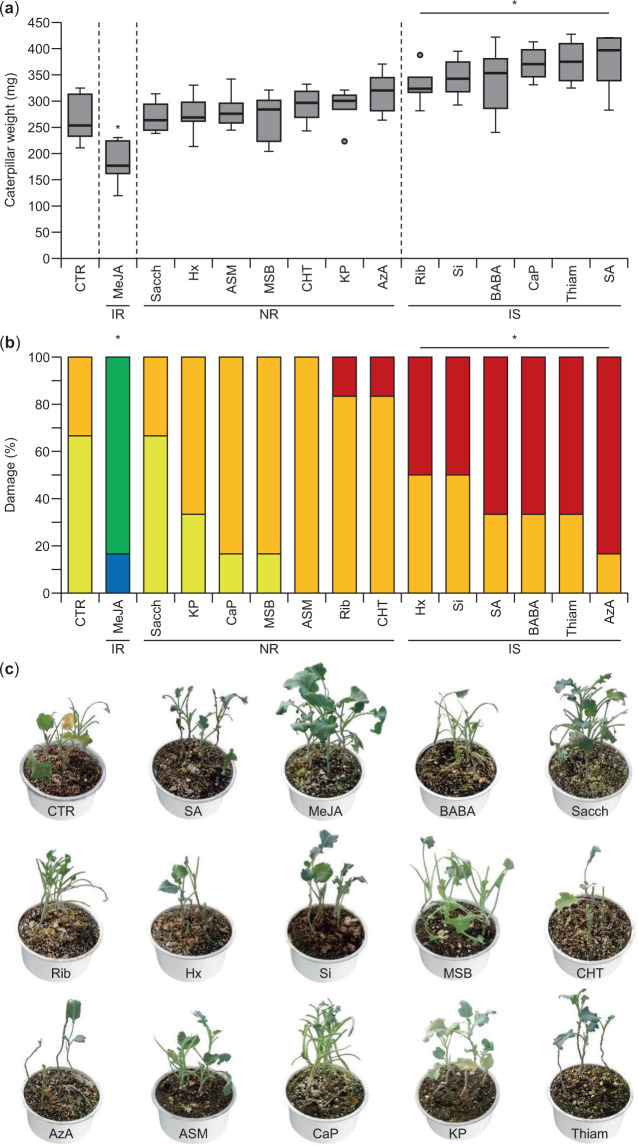
Resistance to *Ascia monuste* infestation after elicitor treatment in broccoli. (**a**) Weight of *A. monuste* caterpillars feeding for 20 days on broccoli plants treated with different elicitors (n = 6 biological replicates). Statistical significance was determined by a Student’s *t*-test (*P < 0.05). (**b**) Damage to broccoli plants infested with *A. monuste* caterpillars for 25 days. Blue, no damage; green, light damage; yellow, moderate damage; orange, severe damage; red, total damage (See damage scale on Supplementary Fig. S1). Statistical significance was determined by a Chi-square test (*P < 0.05). n = 6 biological replicates with four plants per replicate. (**c**) Damage to broccoli plants infested with *A. monuste* caterpillars for 25 days. IR, induced resistance; NR, no response; IS, induced susceptibility.

In addition to the effect of the different elicitor molecules on drought stress tolerance in broccoli, we also evaluated the effect of elicitor treatment on resistance to chewing insects. Pots containing four broccoli plants were treated with the different elicitors (Table 1), and 24 h after elicitor treatment, the plants were infested with *A. monuste* caterpillars. Caterpillar weight was scored 5, 10, 15, and 20 days after elicitor treatment (Table S1–S4; Fig. 3a), and pupal weight (Table S5), pupal transformation time (Table S6), and plant damage (Fig. 3b,c) were measured.

Compared to mock-treated control plants, broccoli treated with MeJA showed increased resistance against feeding *A*. *monuste* caterpillars. Compared to caterpillars feeding on control plants, caterpillars feeding on MeJA-treated plants had a reduced larval and pupal weight (Fig. 3a, Table S1-S5). Furthermore, compared to mock-treated control plants, damage to the leaves was significantly lower in plants treated with MeJA (Fig. 3b,c; Fig. S1). Treatment with the other elicitors led to either no effect on resistance to *A. monuste* infestation or even to increased susceptibility to the feeding caterpillars. The majority of the treatments (Sacch, Rib, Hx, MBS, CHT, AzA, ASM, CaP, and KP) did not influence larval or pupal weight and did not alter plant damage upon *A. monuste* infestation (Fig. 3a-c; Table S1-S5). However, treatment with SA, BABA, Si, or Thiam led to induced susceptibility to *A. monuste* caterpillar feeding. This was reflected by a higher larval and pupal weight and increased damage to the leaves compared to mock-treated control plants. Taken all together, these results suggest that some types of elicitor molecules can effectively induce plant resistance to chewing insects, but, strikingly, those elicitors can induce susceptibility.

### Resistance to hemibiotrophic pathogens after elicitor treatment

**Figure 4:**
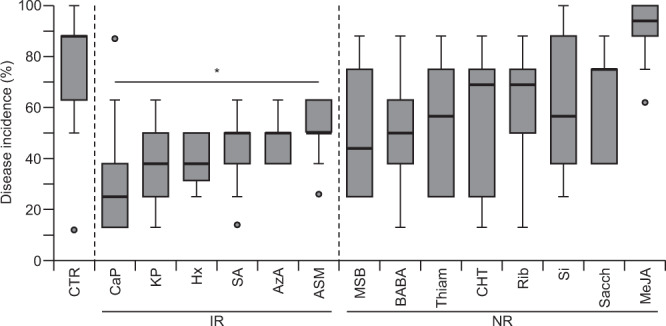
Resistance to the hemibiotrophic bacterium *Pseudomonas syringae* DC3000 after elicitor treatment. Effect of elicitor molecules on disease incidence in *Arabidopsis thaliana* Col-0 plants two weeks after infection with *P. syringae* (n = 10 biological replicates with five plants per replicate). Statistical significance was determined by a Student’s *t*-test (*P < 0.05). IR, induced resistance; NR, no response.

To assess the effect of elicitor treatment on resistance to hemibiotrophic pathogens, we switched to *Arabidopsis*, the model plant for the study of stress resistance that, like broccoli, belongs to the Brassicaceae family. Like for the assays with broccoli plants, elicitors were applied to the growth substrate of 5-week-old *Arabidopsis* Col-0 plants. Twenty-four hours after elicitor treatment, the plants were infected with *Pseudomonas syringae* DC3000, and two weeks after infection with the pathogen, disease incidence was evaluated (Fig. 4). In mock-treated control plants, 74.1% of the leaves showed lesions indicative of pathogen infection. In plants treated with CaP (32.9%), KP (37.8%), Hx (39.25%), SA (45.2%), AzA (47.7%), and ASM (50.2%), disease incidence was significantly reduced. No significant changes compared to mock-treated plants were observed for the other elicitors. However not significant, plants pre-treated with MeJA showed a higher disease incidence (90.2%) compared to the mock-treated plants. These results indicate the potential of several of the tested elicitor molecules to induce resistance to hemibiotrophic pathogens.

### Resistance to necrotrophic pathogens after elicitor treatment

**Figure 5:**
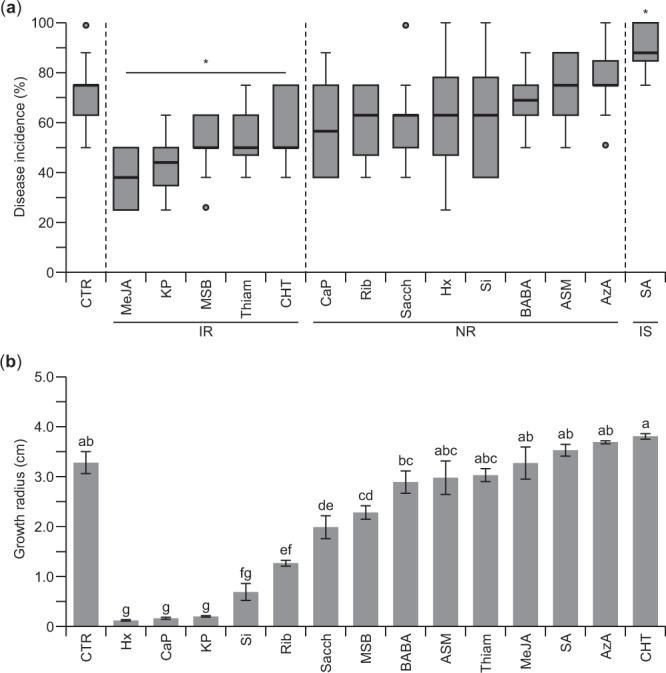
Resistance to the necrotrophic fungus *Alternaria alternata* after elicitor treatment. (**a**) Effect of elicitor molecules on disease incidence in *Arabidopsis thaliana pad-3* mutants one week after infection with *A. alternata* (n = 10 biological replicates with 5 plants per replicate). Statistical significance was determined by a Student’s *t*-test (*P < 0.05). (**b**) Effect of the elicitors on *A. alternata* growth on PDA medium. Colony growth was evaluated 12 days after plating. The error bars designate the standard error (n = 9 technical replicates). The different letters indicate the statistical significance determined by ANOVA (*P* < 0.001) with a post-hoc Tukey test (*P* < 0.05). IR, induced resistance; NR, no response; IS, induced susceptibility.

In addition to resistance to hemibiotrophic pathogens, we also investigated whether elicitor treatment altered resistance of *Arabidopsis* to necrotrophic pathogens. For this assay, we used *Arabidopsis pad-3* mutants as they are susceptible to necrotrophic pathogens and display a clear phenotype upon PAD-3-independent induced pathogen resistance^39^. Like for the experiments with *P. syringae*, 5-week-old *Arabidopsis pad-3* mutants were infected with *Alternaria alternata* 24 h after elicitor treatment. One week after the elicitor application, the disease incidence was scored (Fig. 5a). Compared to mock-treated plants that showed 71.4% of disease incidence, plants pre-treated with SA showed increased susceptibility to *A. alternata* infection, with a disease incidence of 89.0%. Treatments with MeJA (37.7%), KP (42.7%), MSB (51.5%), Thiam (52.7%) and CHT (57.6%) significantly reduced disease incidence compared to the control treatment.

To exclude that the used elicitors act as direct fungicides rather than having antifungal activity through induced plant resistance, we assessed *A. alternata* growth on media containing the different elicitors. Direct effects on the development of *A. alternata* were observed for 6.0 mM Hx, 12.9 mM CaP, 13.0 mM KP, and 166.7 mM Si (Fig. 5b). These results suggest that in addition to triggering the induced defense response of the plant, some of the used elicitors may also exhibit direct fungicidal effects against *A. alternata*.

### Induced upregulation of defense marker genes after elicitor application

**Figure 6:**
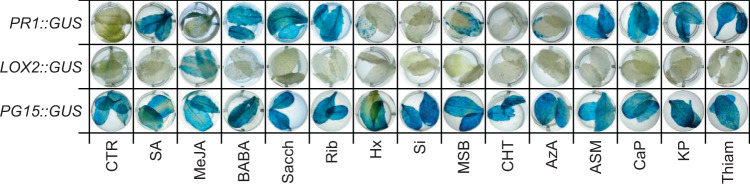
Activation of defense marker genes in the *Arabidopsis thaliana PG15::GUS*, *PR1::GUS*, and *LOX2::GUS* reporter lines after elicitor treatment. Blue color indicates activation of gene expression; white/yellow color represents no gene expression. Similar results were obtained for four biological replicates.

To identify the signaling pathway stimulated by each elicitor molecule, defense marker genes involved in the SA and JA signaling pathways were selected. The upregulation of the *PATHOGENESIS-RELATED GENE 1* (*PR1*) gene is considered a suitable marker for activation of the SA pathway^40^. Similarly, *LIPOXYGENASE 2* (*LOX2*) involved in JA biosynthesis is a suitable marker for the upregulation of the JA pathway^41^. Thus, histochemical GUS assays using the *PR1::GUS* and *LOX2::GUS* reporter constructs were performed on plants treated with the different elicitors (Fig. 6). The *PG15::GUS* line was used as a control for constitutive GUS expression. As expected, SA treatment led to the activation of the *PR1* gene promoter, whereas MeJA treatment led to the activation of the *LOX2* gene promoter. Notably, treatment with eight of the other elicitors (BABA, Sacch, Rib, MBS, ASM, CaP, KP, and Thiam) led to the activation of the *PR1* promoter, indicating these elicitors activated the endogenous defense response via direct upregulation of the SA-mediated signaling pathway. The treatments with Hx, Si, CHT, and AzA did not lead to activation of the *PR1::GUS* or *LOX2::GUS* reporter constructs under our experimental conditions (Fig. 6). As these transgenic *Arabidopsis* lines were not exposed to any stress after elicitor application, the upregulation of the marker genes indicates activation of the direct activation of defense response by the elicitors. Maybe the absence of GUS expression after the elicitor application could be explained that treated plants are yet in a primed state, that will be activated after stress.

### SA is involved in drought stress tolerance in *Arabidopsis*

**Figure 7:**
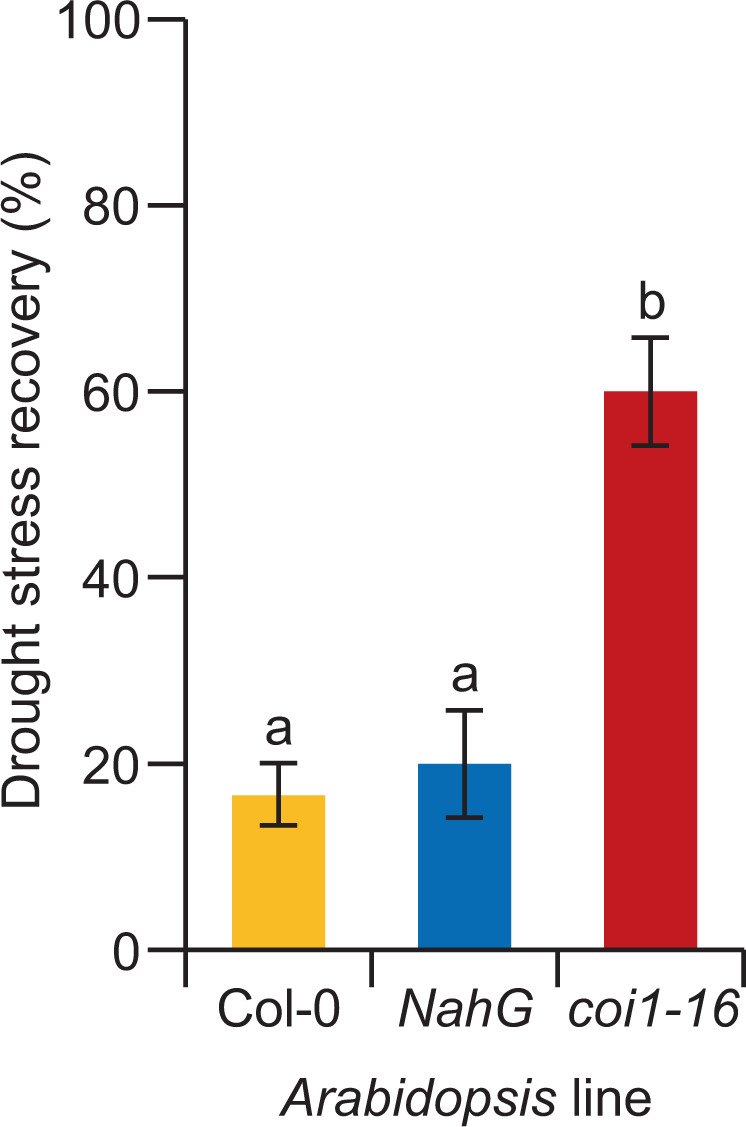
SA is involved in drought stress tolerance. Drought stress recovery (in %) of the *Arabidopsis thaliana* Col-0 (wild-type), NahG (SA deficient), and *coi1–16* (JA pathway mutant with enhanced SA levels) lines. The error bars designate the standard error (n = 3 biological replicates with ten plants per replicate). The different letters indicate the statistical significance determined by ANOVA (*P* = 0.002) with a post-hoc Tukey test (*P* < 0.05).

In the drought stress assays with broccoli plants, all applied elicitors, except MeJA, led to a similar enhanced drought tolerance as treatment with SA. Furthermore, activation of the *PR1::GUS* reporter construct by SA and the majority of the elicitor molecules was observed. Together these data suggest that the SA signaling pathway is crucial for the regulation of drought tolerance responses. To corroborate the role of SA in drought recovery responses, drought stress experiments were carried out with wild-type *Arabidopsis* Col-0 plants and two lines with altered SA accumulation. The *Arabidopsis* NahG transgenic line expresses a salicylate hydroxylase that converts SA to catechol, leading to plants depleted of SA^42^. The *coi1–16* mutant line, on the other hand, has an impaired JA signaling, which leads to endogenous overaccumulation of SA^43^. Drought stress assays with these lines revealed that the NahG transgenic line had a similar drought stress recovery as wild-type Col-0 plants (20% recovery). However, the *coi1–16* mutant line with enhanced SA accumulation showed increased drought stress tolerance, with up to 60% recovery after stress application (Fig. 7). These results corroborate the notion that endogenous SA plays an important role in triggering plant tolerance to drought conditions (Figs. 1, 2).

### SA influences water potential (*Ψ*_w_), stomata conductance (gs), and chlorophyll fluorescence (Fv/Fm), leading to enhanced drought tolerance

**Figure 8:**
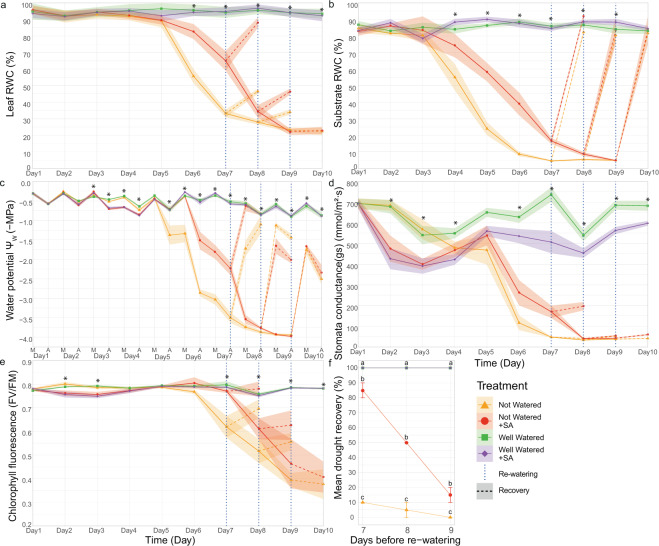
Physiological responses to drought stress of broccoli plants treated with salicylic acid (SA). 14-day-old broccoli plants were SA-treated or mock-treated and exposed to drought conditions for nine days, while another group of SA-treated or mock-treated plants were continuously well-watered. From every treatment, at day seven, eight, and nine, a group of plants were re-watered (vertical blue dotted line), and recovery was assessed one day later (dashed line). (**a**) Relative water content (RWC) of the leaves (n = 10 per day), (**b**) relative water content of the substrate (n = 10 per day), (**c**) plant water potential Ψw (n = 5 of each time point), where M means morning and A means afternoon measurements (**d**) stomata conductance gs (n = 10 per day), and (**e**) chlorophyll fluorescence FV/FM (n = 10 per day) (See materials and methods for details). The solid line represents the mean, and the ribbon shows the mean standard error. *Significant difference (ANOVA; *p* < 0.05) Supplementary Table S7 includes the post-hoc Tukey HSD test for each time point. (**f**) Drought recovery after re-watering (n = 20 plants per treatment). Each point represents the mean, and the bars indicate the standard error. Two biological replicates were performed of the entire experiment. The different letters indicate the statistical significance determined by ANOVA (P < 0.001) with a post-hoc Tukey test (P < 0.05).

To gain insight into the mechanism leading to SA-mediated induced drought tolerance, we measured the drought recovery and relative water content (RWC) of both the leaves and the growth substrate of SA-treated and mock-treated broccoli plants. As expected and in contrast to plants under constant irrigation, the RWC of both leaves and growth substrate was reduced due to water loss when water was withheld (Fig. 8a,b). In accordance with our previous experiments, five to nine days after water was withheld, the RWC of SA-treated plants was significantly higher than the RWC of control plants (Fig. 8a). Interestingly, also the RWC of the growth substrate was higher in SA-treated plants (Fig. 8b), indicating that reduced water loss was reflected in reduced water uptake from the soil. Furthermore, seven, eight, and nine days after water was withheld, a subset of the plants was re-watered (Fig. 8, vertical blue dotted line), and their drought recovery was evaluated the following day (Fig. 8, dashed lines). On day 7, SA-treated plants of which water was withheld showed less visual symptoms of drought stress (Supplementary Fig. S2) and increased recovery of up to 85% on day 8, compared to mock-treated control plants of which only 10% recovered (Fig. 8f). After 8 and 9 days of drought, the recovery of SA-treated plants decreased to 50% and 20%, respectively. However, recovery was always significantly higher compared to mock-treated plants. Together, these results indicate that SA protects the plant from water loss, which leads to reduced water uptake from the soil, thereby delaying the effects of the applied drought stress.

To investigate the mechanisms that lead to reduced water loss upon treatment with SA, we monitored the water potential (*Ψ*w), stomata conductance (gs), and chlorophyll fluorescence (Fv/Fm) of the plants during this experiment (Fig. 8c–e). Three to five days after elicitor treatment, the water potential of SA-treated plants was slightly, but significantly, reduced, both in well-watered control conditions and in drought conditions (Fig. 8c), compared to mock-treated control plants. Five days after water was withheld, a drastic reduction of water potential was observed in the mock-treated control plants, and these plants were the first to reach the lowest water potential value (close to −4 MPa; Fig. 8c). Thus, SA treatment leads to an initial slightly lower water potential, but compared to the control plants, a drastic drop in water potential due to drought stress was delayed. Furthermore, significantly lower gs values of SA-treated plants both in well-watered control conditions and in drought conditions (Fig. 8d) indicated that the application of SA induced stomata closure within the day. Stomata closure in SA-treated plants may reduce water evaporation from the leaves and lead to lower water potential. Consequently, less water is taken up from the soil, leading to a delayed onset of drought stress. Besides, we also observed a reduction in chlorophyll fluorescence in SA-treated plants, both in well-watered control conditions and in drought conditions, at day 2 and 3 of the experiment (Fig. 8e), indicating reduced activity of photosystem II (PSII) upon SA treatment. Later, when the drought stress became critical, chlorophyll fluorescence dropped sharply. Like for the reduced water potential, this sharp drop was observed first for the mock-treated control plants.

## Discussion

To understand the effect of elicitor molecules in plant defense, we performed experiments with two plant species from the Brassicaceae family, the globally produced, commercial crop broccoli, and the model plant *A. thaliana*. The reasoning behind our plant selection falls on the premise that the regulation of defense pathways appears to be conserved among the plant kingdom^44^. Therefore, knowledge obtained through the thorough analysis of model plants should be translatable into related commercial crops and *vice versa*.

In this study, fourteen elicitor molecules were tested, and their effects against different types of abiotic and biotic stresses were determined. Table 2 summarizes our findings on the relationships between elicitor molecules, upregulation of defense signaling pathways, and induction of resistance/susceptibility. These results confirm previous findings where the application of chemical molecules effectively induces defense mechanisms in plants^2,19^. Nevertheless, we observed that elicitors could stimulate certain specific, but not all defense responses to diverse kinds of plant stresses (Table 2).

**Table 2:** Overview of the effects of the different elicitor molecules on the applied stresses.

Elicitor	Abiotic stress (Drought stress)		Hemibiotrophic bacterium (*P. syringae*)	Necrotrophic fungus (*A. alternata*)	Chewing insect (*A. monuste*)	SA-dependent gene (PR1::GUS)	JA-dependent gene (LOX2::GUS)
SA	IR	Medium	IR	IS	IS	Induced	NR
MeJA	NR	NR	NR	IR	IR	NR	Induced
BABA	IR	Long	NR	NR	IS	Induced	NR
Sacch	IR	Long	NR	NR	NR	Induced	NR
Rib	IR	Short	NR	NR	NR	Induced	NR
Hx	IR	Short	IR	NR	NR	NR	NR
Si	IR	Long	NR	NR	IS	NR	NR
MSB	IR	Short	NR	IR	NR	Induced	NR
CHT	IR	Short	NR	IR	NR	NR	NR
AzA	IR	Medium	IR	NR	NR	NR	NR
ASM	IR	Short	IR	NR	NR	Induced	NR
CaP	IR	Short	IR	NR	NR	Induced	NR
KP	IR	Short	IR	IR	NR	Induced	NR
Thiam	IR	Short	NR	IR	IS	Induced	NR

Abbreviations: IR: induced resistance; IS: induced susceptibility, NR: not responsive.

### Induced resistance to abiotic stresses

Our results revealed that broccoli plants pre-treated with all selected elicitors, except MeJA, showed increased recovery from drought stress compared to mock-treated plants (Fig. 1). Furthermore, drought tolerance induction was depending on the concentration of the elicitor molecule (Fig. 1). Interestingly, most of the applied elicitors activated the SA-dependent signaling pathway (Fig. 6); and *Arabidopsi*s *coi1–16* mutants were less affected by drought stress compared to wild-type or NahG lines with normal or lower SA levels (Fig. 7), suggesting that accumulation SA is crucial for drought stress tolerance in Brassicaceae. These results are in accordance with previous studies that indicated that SA is an essential component of the defense signaling pathway during abiotic stresses such as osmotic stress, salt stress, temperature changes, and toxic metals^45^. Additionally, SA was shown to enhance drought tolerance by regulating metabolic processes such as regulation of stomata, photosynthesis, carbohydrate metabolism, signal transduction, and production of osmolytes and specialized metabolites^46^. In accordance, our results also indicate that the induced drought tolerance of SA-treated plants was associated with stomata control. After SA treatment, we observed decreased stomatal conductance, reflective of stomata closure, and a reduction of the water potential of the plants. Together, these factors may lead to a reduced water uptake from the soil, leading to a soil that is longer hydrated and longer survival of the plants in drought conditions. These observations are also following previous studies that demonstrated that the application of SA in diverse plant species alleviates drought stress by adjusting water potential, stomatal conductance, and transpiration rate^47–50^ and leads to reduced adverse effects of drought on photosynthesis activity^47,48,51^. These physiological changes in SA-treated plants might allow them to prepare defensive measures against the oncoming drought stress. Overall, these data implicate that the SA-dependent pathway at least in part regulates induced resistance to drought stress. Nevertheless, most of the previous research on SA focused on defense responses against pathogens, leading to an incomplete understanding of the role of SA in abiotic stress and warranting a more thorough molecular dissection to reveal the role of SA in abiotic stress.

Furthermore, induced drought tolerance was observed up to three weeks after the elicitor application (Fig. 2). Although the stability of induced resistance over time is not yet well understood, and related knowledge is scarce, it might constitute a key element for future research to enable the widespread use of elicitor molecules in agriculture. A previous study shows the persistence of induced resistance against *Colletotrichum lagenarium* in cucumber plants during six weeks^52^, although the molecular mechanisms leading to the induced resistance remain to be uncovered. Alternatively, it has been suggested that the persistence of the enhanced state of resistance caused by biotic or abiotic factors can be explained by the priming mechanism. Primed plants can accelerate the synthesis and accumulation of elements needed for the activation of resistance, including transcription factors that lead to a faster and stronger activation of defense genes, signaling pathways and physiological mechanisms leading to induced resistance^14,53^. Our results indicate an enhanced persistence of induced resistance by application of elicitor molecules. However, future research is required to uncover the molecular mechanisms behind this phenomenon.

### Induced resistance to biotic stresses

The induced resistance to the hemibiotrophic pathogenic bacterium *P. syringae* was effectively induced by treatment with SA, CaP, KP, Hx, AzA, or ASM (Fig. 3). Previously, the application of phosphites was reported to induce defense responses against biotrophic pathogens. For instance, treatment with phosphites led to increased resistance of *Arabidopsis* towards *P. syringae* and *Hyaloperonospora arabidopsidis*, via the SA-dependent signaling pathway^54^. Also, in our study, we observed that the application of SA or the phosphite salts CaP and KP led to increased resistance to *P. syringae* by triggering of the SA-dependent signaling pathway. This observation is in agreement with other studies^6,7,54–56^ and indicates a robust induced resistance towards biotrophic pathogens by triggering the SA-signaling pathway through the application of SA or phosphites.

Effective resistance responses against the necrotrophic fungus *A. alternata* were observed after the application of MeJA, KP, MSB, Thiam, and CHT. Conversely, SA induced susceptibility (Fig. 5). MeJA, which activates the JA-signaling pathway, is a well-known activator of the defense signaling pathway against necrotrophic pathogens^6,57,58^. Moreover, KP was shown to be an effective elicitor molecule for the control of necrotrophic pathogens as it leads to the accumulation of pectin in cortical tissues and an increased content and/or activity of polygalacturonase and proteinase inhibitors^59,60^. Likewise, K^+^ starvation was shown to lead to increased transcription of JA biosynthesis genes and JA-responsive genes^61^. Besides, some studies mention the potential of the use of other elicitors such as MSB against necrotrophic pathogens^30^, however, they were not significantly different in our experimental conditions. Here, our data demonstrate that the use of certain elicitor molecules can induce effective responses against necrotrophic pathogens.

Some inducers can display a direct toxic effect against a wide range of attackers when applied directly on the pathogen. We observed direct inhibitory effects during the *in vitro* growth of *A. alternata* when Hx, CaP, or KP were added to the growth medium (Fig. 5b). The fungicidal properties of Hx have been reported previously^62^. The double property as inductor of resistance and as a fungicide entails promising use in future plant protection practices. Nevertheless, in our study, all fourteen elicitors were applied to the roots to exploit the systemic induced resistance throughout the plant and exclude the fungicidal effects. In line with our findings, it is known that belowground defense stimulation can effectively cause aboveground defenses^63^. Altogether, our data demonstrate that resistance responses observed aboveground by the application of elicitors in the soil were systemic. However, effectiveness between soil versus foliar applications has to be explored in the future.

Effective induced resistance against the herbivorous chewing insect *A. monuste* was only achieved by MeJA treatment (Fig. 3). The application of MeJA led to a reduced caterpillar weight and, consequently, their survival likelihood. In agreement with previous reports^6,64–66^, it is evident that MeJA stimulated the JA signaling pathway, leading to the activation of the JA-mediated defense mechanisms. An increased insect resistance could lead to lower costs for pest control for commercial broccoli cultivation. Nevertheless, more studies to find cost-effective JA-dependent elicitor molecules and their respective doses to activate defense responses against insects are required.

Interestingly, besides the increased resistance observed after MeJA treatment, four of the applied elicitor molecules led to enhanced susceptibility to *A. monuste*, reflected by increased caterpillar weight and damage to the plants (Fig. 3). In contrast to the many studies that demonstrate that JA application contributes to induced resistance against herbivorous insects and necrotrophic pathogens, SA was repeatedly shown to increase susceptibility to these biotic stresses^6,15,67–69^, which is in line with the results found in this study (Figs. 3, 5). More specifically, the activation of defense responses against chewing insects such as Lepidoptera and cell-content feeders such as thrips are mediated by the JA-signaling pathway^57,70^. At the same time, these defense responses have an antagonistic effect on the defense responses to phloem-feeders such as aphids and biotrophic pathogens that are controlled by the SA-pathway^71^. Nine of the applied elicitor molecules induced the SA defense pathway (Fig. 6), which may lead to antagonistic effects on the JA defense pathway, and, consequently, enhanced susceptibility to herbivorous insects and necrotrophic pathogens. It was reported before that the activation of the SA pathway suppresses resistance and expression of defense responses related to the JA pathway^6,56,72,73^. Similarly, several elicitor agents were shown to induce systemic susceptibility^74^. Thus, elicitor molecules stimulating the JA-pathway can induce resistance against chewing insects and necrotrophic pathogens, whereas, elicitor molecules that exploit the SA-pathway can induce susceptibility to these biotic stresses. Cross-talk between the two pathways is an important item to take into account in integrated pest management programs as the use of an elicitor molecule against a certain type of stress may actually lead to increased susceptibility towards another stress.

### Elicitors modulate hormone-related responses

Finally, based on all the analyses we classified the applied elicitor molecules in three groups: (i) compounds that lead to direct induction of resistance mediated by the SA signaling cascade; (ii) compounds that might lead to resistance independent of the SA or JA signaling cascade or priming; and (iii) compounds that lead to the direct induction of resistance mediated by the JA signaling cascade. Elicitors belonging to the first group stimulated drought stress tolerance, induced resistance against hemibiotrophic pathogens, and led to the direct upregulation of the *PR-1* gene. However, they also led to increased susceptibility to *A. monuste* feeding. The elicitor molecules classified in this group are SA, BABA, Sacch, Rib, MSB, ASM, CaP, KP, and Thiam.

Elicitor molecules of the second group did not induce direct expression of any of the tested defense marker genes. However, they were effective in inducing tolerance to drought stress. This resistance effect might be attributed to mechanisms independent of SA, JA, or induction of the priming effect. As already indicated before, priming leads to more robust and faster activation of defense responses when a threat is present^53^. However, in the absence of the stress, responses might not be detected since plant defense is not activated yet. Nevertheless, compared to the mock treatment, treatments with compounds belonging to the second group showed similar or even lower susceptibility to the necrotrophic fungus *A. alternat*a. Furthermore, they presented similar or increased levels of susceptibility to the Lepidoptera *A. monuste*. Hence, these elicitors did not induce the priming effect. The elicitor molecules belonging to this group are Hx, Si, CHT, and AzA. Although it has been reported that CHT and Hx activate the JA signaling pathway^27,75^, we did not observe this under our experimental conditions. Hence, a change in environmental conditions may lead to a different output of plant responses^76^, and thus a more thorough assessment of their tolerance mechanisms and their connection with priming or SA- and JA- mediated defenses in different environmental conditions is warranted.

The third group contains only one metabolite, MeJA. MeJA induced the expression of the JA-dependent *LOX2* gene (Fig. 6), and thus the direct activation of the JA defense pathway^56,58^. MeJA application induced systemic defense against the chewing insect *A. monuste*, reflected by a delayed insect development and a drastically reduced damage to the broccoli plants (Fig. 3), and to the necrotrophic fungus *A. alternata*, reflected by a lower disease incidence (Fig. 5a). As already indicated above, the immune responses against chewing insects mediated by the JA-pathway have been reported in various studies^6,64–66^. Strikingly, JA-induction might induce an antagonist effect on the SA-mediated pathway^77^. In this study, the only treatment inducing the JA-pathway was MeJA; therefore, the identification of additional chemical elicitors capable of generating the JA-mediated defense responses is required.

In conclusion, our results suggest that most of the tested elicitor molecules exploit the SA-mediated defense responses. On the one hand, induction of the SA signaling can lead to the activation of defense responses to alleviate abiotic stress such as drought and defense against hemibiotrophic pathogens. On the other hand, however, this might also lead to the repression of the JA-mediated defense pathways and increased susceptibility to necrotrophic pathogens and chewing insects. Future studies must evaluate a greater variety of elicitor molecules, and under more diverse experimental conditions (temperature, light, nutrient availability among others), since different elicitor molecule concentrations can modulate the activation of defense pathways in different ways^78^. Although the mechanisms of elicitor molecules to induce defense responses are still not completely understood, the results found in this study may contribute to a clearer and extensive description of their performance on several types of plant stress to optimize their future use as an effective tool for plant protection.

## Materials and Methods

### Plant material and growth conditions

The experiments were performed with broccoli plantlets (*Brassica oleracea* L. var. *italica* cv. Legacy; PilvicSA, Latacunga, Ecuador) and the *A. thaliana* Col-0, NahG^42^, *coi1–16*^79^, *pad-3*^39^, *PG15::GUS*, *PR-1::GUS*, and *LOX2::GUS*^72^ lines. Plants were grown under greenhouse conditions with a 12 h/12 h light/dark regime and a temperature ranging from 14 °C to 25 °C. Details of the plant growth conditions for each experiment are described below.

### Elicitor molecules

The elicitor molecules were purchased from Sigma-Aldrich, Saint Louis, MO, USA (SA, MeJA, BABA, Sacch, Rib, Hx, Si, MSB, CHT, AzA, Thiam); Syngenta, Basel, Switzerland (ASM) or Daymsa, Zaragoza, Spain (CaP, KP). The concentrations that were used for the elicitor molecules are indicated in Table 1 and were chosen according to the concentrations reported in the publications referred to in Table 1. For all experiments, the treatments were applied to the substrate containing the roots.

### Drought tolerance assays in broccoli and *Arabidopsis*

Drought tolerance assays to screen the different elicitor molecules were performed with a method adapted from^80^. Under greenhouse conditions, 10 ml of each elicitor solution was poured into an 85-ml plastic pot containing a 14-day-old broccoli plantlet grown in peat. Next, the plants were deprived of water until a relative water content (RWC) of 0.30 was reached (7 to 8 days period). The RWC was monitored using additional mock samples and was calculated as proposed^81^, using the formula:

1 $$RWC=\frac{FW-DW}{TW-DW}$$

First, the fresh weight (FW) was determined by weighing the leaves immediately after detaching them from the plant. Next, the detached leaves were incubated in distilled water for four h at room temperature under normal light, after which they were briefly dried, and the turgid weight (TW) was recorded. Finally, the leaves were dried for two days in an oven at 80 °C, after which the dry weight (DW) was measured.

After the drought period, the plants were re-watered using 10 ml of distilled water, and drought stress recovery was evaluated after 24 h. One biological replicate consisted of 10 plants. The number of tolerant (showing turgid leaves) and susceptible (showing withered leaves) plants was assessed visually and expressed as a percentage per biological replicate. Five biological replicates were performed, each one in a different week. For broccoli, three different concentrations for each elicitor were tested: the optimum concentration reported in the literature and 3-fold reduced and 3-fold increased concentrations (Table 1). A mock treatment consisting of distilled water was included as a control.

Additionally, a drought experiment was performed with *Arabidopsis* genotypes to test the hypothesis that the SA-signaling pathway is crucial for the induction of drought tolerance responses, according to findings from drought screening (Fig. 1) and upregulation of defense marker genes (Fig. 6). This drought experiment followed a similar procedure as described above using five-week-old *Arabidopsis* plants, but instead of elicitor molecules, the following genotypes were employed: Col-0 (wild-type), NahG (lack of SA accumulation) and *coi1–16* (JA deficient mutant). One biological replicate consisted of 10 plants. Three biological replicates were performed, each one in a different week.

### Persistence of drought tolerance assay in broccoli

The persistence of the induced tolerance to drought stress was measured by challenging the plants with drought stress one, two, or three weeks after the elicitor application. For this experiment, the best concentration of each elicitor molecule obtained from the drought tolerance experiment was tested (Table 1), and a similar method was used. The plants were divided into different groups after the elicitor application, and the drought stress was applied in each group, either one, two, or three weeks after the elicitor application. Before the drought stress, the plants were regularly watered with 10 ml of half-strength Hoagland solution. For the drought stress, each set of broccoli plants was deprived of water until reaching an RWC of 0.30. Next, the plants were re-watered, and after 24 h, the evaluation of tolerant and susceptible plants was performed. One biological replicate consisted of 10 plants. The results were expressed as a percentage of drought recovery. Five biological replicates were performed, each one in a different week.

### Chewing insect resistance assay in broccoli

The chewing insect resistance assay in broccoli was performed according to^82^ with modifications. 14-day-old broccoli plants were transferred to 500-ml plastic pots containing a substrate composed of peat and perlite (1:1). The plants were watered every week with half-strength Hoagland solution. After 21 days, the plants were treated with 200 ml of elicitor solution (Table 1). The next day, one *A. monuste* (Lepidoptera: Pieridae) caterpillar was placed onto each pot of the plants. To assure homogeneous larvae, they were selected from the same egg batch when they molted to their second instar with an approximate weight of 5 mg. The caterpillar weight (5, 10, 15, and 20 days after infestation), pupae weight, and pupae transformation time were monitored. To weigh the caterpillars, they were individually transferred to a 50-ml plastic falcon tube, weighed, and finally returned to the original plant. Additionally, plant damage was measured visually 25 days after caterpillar infestation by visually comparing the plant with a damage scale consisting of five damage degrees: no damage, light, moderate, severe, and total damage (Fig. S1). One biological replicate consisted of a pot containing four broccoli plants and one caterpillar. Additional biological replicates were included in each experiment to compensate for deceased caterpillars. Six biological replicates were used for the statistical analysis; two replicates were performed, each one in a different week.

### Biotrophic pathogen resistance assay in *Arabidopsis*

For this experiment, five-week-old *A. thaliana* Col-0 plants were pre-treated with different elicitor molecules (Table 1). Twenty-four hours later, they were infected with *P. syringae* pv. tomato DC3000 by dipping the plants into a *P. syringae* solution containing 2.5 × 10^7^ cfu/ml. The disease incidence was evaluated after two weeks. The eight bigger and older leaves were assessed by counting the number of leaves showing lesions (presence of water-soaked lesions and chlorosis) as described^83^ and expressed as a percentage. One biological replicate consisted of five plants. Ten biological replicates were used.

### Necrotrophic pathogen resistance assay in *Arabidopsis*

A suitable pathosystem to study induced resistance to necrotrophic pathogens consists of *A. thaliana pad-3* mutants that are impaired in camalexin biosynthesis and susceptible to necrotrophic pathogens^39^. Five-week-old *Arabidopsis pad-3* plants were pre-treated with elicitor molecules (Table 1). Twenty-four hours later, they were inoculated with the necrotrophic fungus *A. alternata* by applying 3 µl droplets of a suspension containing 5 × 10^5^ conidia/ml on the eight bigger and older leaves. The disease incidence was evaluated one week after infection as described^84^, reporting the number of leaves with lesions as percentages. One biological replicate consisted of five plants. Ten biological replicates were used for the analysis.

### *In vitroA. alternata* growth measurement on medium supplemented with elicitors

The fungus *A. alternata* was cultured in Petri dishes with PDA medium containing different concentrations of each elicitor molecule (Table 1), according to^85^. The fungal growth radius was measured six and twelve days after inoculation. One biological replicate consisted of one Petri dish containing one cm^2^ of the pathogen on the PDA medium. Nine biological replicates were used for this experiment.

### GUS histochemical assays in *Arabidopsis* reporter lines

Seeds of the *A. thaliana PG15::GUS*, *PR1::GUS* and *LOX2::GUS* reporter lines were sterilized for three h with chlorine gas and sown on plates containing Murashige and Skoog (MS) medium (pH 5.7) with 2% sucrose and 0.8% plant tissue culture agar. Fourteen days after germination, seedlings were rinsed with distilled water and placed in a culture dish well to which the elicitors were added (Table 1). Twenty-four hours later, the plants were harvested, and the histochemical GUS assay was performed as described^72^. One biological replicate consisted of five plants. Four biological replicates were used for this analysis.

### Physiological response to drought assay in broccoli

A drought experiment on 14-days-old broccoli plants was performed to assess several physiological parameters over ten days daily. The physiological parameters measured were: the relative water content (RWC) of the leaf, the RWC of the growth substrate, drought recovery of the plants, water potential (Ψw), stomata conductance (gs), and chlorophyll fluorescence (Fv/Fm). For this experiment, SA-treated and mock-treated plants were subjected to well-watered and no water conditions. The experiment was conducted similarly to the drought tolerance assays in broccoli (Materials and Methods section 4.3), but different groups of plants were re-watered 7, 8, and 9 days after water was withheld. One biological replicate consisted of a set of 10 plants per treatment per day, and two biological replicates were performed, showing similar results.

Plant water potential (Ψw) was measured two times a day, between 6 and 7 am, and between 4 and 5 pm. The whole aerial part (leaves and stem) of the five broccoli plants was used for measurements for each treatment and time point. Ψw was measured with a Scholander pressure chamber (PMS Model 615D, Fresno, CA, USA) as described^86,87^. For each time point, five samples per treatment were measured.

Leaf stomatal conductance (gs) measurements were taken with a steady-state diffusion porometer (Model SC-1, Decagon Devices, USA) calibrated by the manufacturer. The porometer clamp was placed on the second true leaf of each broccoli plant. Measurements were conducted from 10 am to 2 pm, when the stomata are open. Every day, ten plants were measured per treatment. A stabilization period (5 to 10 min) of the leaf porometer was required before each measurement. As described^88^, all gs measurements were obtained from leaves that were fully exposed to the sunlight. Ten plants were measured per day per treatment.

For chlorophyll fluorescence measurements, dark adapting clips were placed on the second true leaf of each broccoli plant and left for 60 min for dark adaptation. After this period, Fv/Fm was measured using an OS30p + modulated fluorometer (Opti-Sciences Inc, New Hampshire USA) as described^89^. Five plants were measured at 7 am, and another five plants were measured at 4 pm, and the average Chlorophyll fluorescence measurements of the ten plants were plotted.

### Statistical analysis

Analysis of variance (ANOVA) with a post-hoc Tukey’s test was used for statistical analysis of the drought stress recovery experiments of broccoli and *Arabidopsis*, the persistence to drought stress of broccoli, the *A. alternata* growth assays, the relative water content (RWC) of the leaf and growth substrate, the water potential (Ψw), the stomata conductance (gs), and the chlorophyll fluorescence (Fv/Fm). The data on caterpillar weight, plant damage to chewing insects, and disease incidence to *P. syringae* and *A. alternata* were subjected to a Student’s *t*-test. For both types of statistical analysis, the data were first tested for normal distribution and equality of variances. All statistical analyses were performed with the IBM SPSS Statistics for Windows version 19 software package (IBM Corp., Armonk, N.Y., USA).

## Supplemenatry information

Supplemenatry information.
