# Supplementary material for: Induced tolerance to abiotic and biotic stresses of broccoli and Arabidopsis after treatment with elicitor molecules
Source: Sci Rep. 2020 Jun 25;10:10319. doi: 10.1038/s41598-020-67074-7 (PMC7316721; doi:10.1038/s41598-020-67074-7)
Supplement: Supplementary file 1 — Supplemenatry information. [file 41598_2020_67074_MOESM1_ESM.docx]

**SupplementaRY materials**

**Induced tolerance to abiotic and biotic stresses of broccoli and *Arabidopsis* after treatment with elicitor molecules**

Jhon J. Venegas-Molina, Silvia Proietti, Jacob Pollier, Wilson Orozco-Freire, Darío Ramírez-Villacis, and Antonio Leon-Reyes

The following supplementary materials are available for this article:

**SUPPLEMENTARY FIGURES:**

**Figure S1.** Representation of the scale of damage of broccoli plants infested with *A. monuste* caterpillars for 25 days.

**Figure S2.** Time course experiment of mock and SA-treated broccoli plants under drought stress after 9 days.

**SUPPLEMENTARY TABLES:**

**Table S1.** Caterpillar weight five days after elicitor treatment.

**Table S2.** Caterpillar weight ten days after elicitor treatment.

**Table S3.** Caterpillar weight fifteen days after elicitor treatment.

**Table S4.** Caterpillar weight twenty days after elicitor treatment.

**Table S5.** Pupal weight.

**Table S6.** Pupal transformation time.

**SUPPLEMENTARY FIGURES**


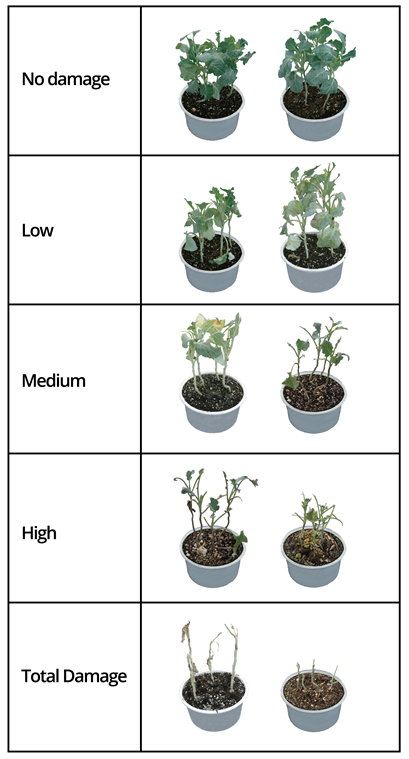


**Figure S1.** Representation of the scale of damage of broccoli plants infested with *A. monuste* caterpillars for 25 days. No damage (91 to 100% of leaves), low damage (71 to 90 % of leaves); medium damage (41 to 70% of leaves); high damage (11 to 40% of leaves) and total damage (0 to 10% of leaves) ranges are presented.


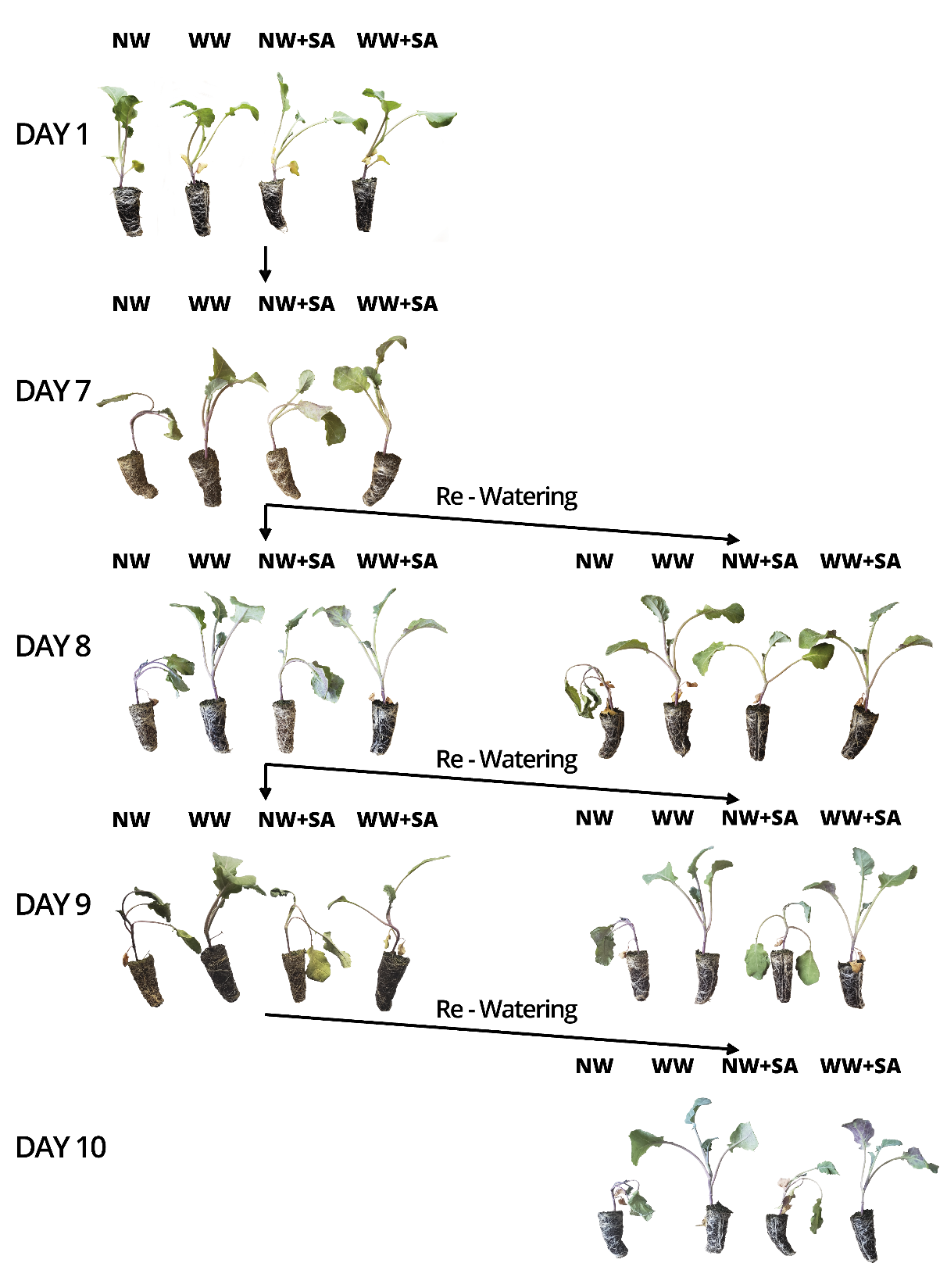


**Figure S2.** Time course experiment of mock and SA-treated broccoli plants under drought stress after 9 days. A set of plants were re-watered at the end of days 7, 8, and 9 for the evaluation of plant recovery.

**SUPPLEMENTARY TABLES**

**Table S1.** Caterpillar weight five days after elicitor treatment.

| **Treatment** | **Caterpillar weight (mg)** | **Standard deviation** |
| --- | --- | --- |
| Sacch | 5.23 | 0.27 |
| Hx | 5.18 | 0.40 |
| CHT | 5.16 | 0.27 |
| Rib | 5.15 | 0.24 |
| SA | 5.15 | 0.41 |
| BABA | 5.10 | 0.26 |
| Thiam | 5.06 | 0.31 |
| CaP | 5.06 | 0.17 |
| AzA | 5.01 | 0.32 |
| CTR | 5.01 | 0.33 |
| ASM | 5.00 | 0.26 |
| MeJA | 4.95 | 0.43 |
| KP | 4.90 | 0.22 |
| Si | 4.81 | 0.43 |
| MSB | 4.78 | 0.27 |

**Table S2.** Caterpillar weight ten days after elicitor treatment.

| **Treatment** | **Caterpillar weight (mg)** | **Standard deviation** |
| --- | --- | --- |
| BABA | 56.03 | 19.16 |
| SA | 42.33 | 16.68 |
| Rib | 41.17 | 17.81 |
| CaP | 41.12 | 20.98 |
| ASM | 40.93 | 18.02 |
| Thiam | 35.10 | 16.29 |
| Sacch | 32.72 | 10.93 |
| AzA | 32.47 | 15.32 |
| Hx | 32.47 | 13.53 |
| Si | 31.87 | 16.29 |
| KP | 31.03 | 18.76 |
| CHT | 28.27 | 13.94 |
| CTR | 24.60 | 3.90 |
| MSB | 22.95 | 8.08 |
| MeJA | 18.12 | 4.75 |

**Table S3.** Caterpillar weight fifteen days after elicitor treatment.

| **Treatment** | **Caterpillar weight (mg)** | **Standard deviation** |
| --- | --- | --- |
| SA | 105.80 | 12.58 |
| Rib | 95.37 | 31.72 |
| Si | 91.68 | 21.37 |
| Thiam | 91.63 | 20.38 |
| CaP | 90.00 | 19.91 |
| AzA | 84.10 | 17.71 |
| BABA | 82.45 | 23.17 |
| Hx | 79.67 | 16.86 |
| Sacch | 71.75 | 8.88 |
| CHT | 71.72 | 16.99 |
| BTH | 71.42 | 20.93 |
| KP | 70.98 | 17.99 |
| MSB | 68.70 | 15.42 |
| CTR | 65.18 | 8.84 |
| MeJA | 50.83 | 8.79 |

**Table S4.** Caterpillar weight twenty days after elicitor treatment.

| **Treatment** | **Caterpillar weight (mg)** | **Standard deviation** |
| --- | --- | --- |
| Control | 264.92 | 50.41 |
| MeJA | 181.83 | 38.25 |
| Sacch | 269.97 | 28.55 |
| MBS | 270.07 | 35.50 |
| Hx | 273.42 | 60.11 |
| BTH | 282.28 | 33.49 |
| Kphi | 290.87 | 37.55 |
| CHT | 292.70 | 30.28 |
| AzA | 316.95 | 30.50 |
| Rib | 330.60 | 31.47 |
| Si | 344.30 | 35.70 |
| BABA | 339.58 | 42.27 |
| CaPhi | 371.78 | 27.48 |
| Thiam | 375.15 | 41.14 |
| SA | 376.17 | 37.51 |

**Table S5.** Pupal weight.

| **Treatment** | **Pupal weight (mg)** | **Standard deviation** |
| --- | --- | --- |
| Thiam | 323.24 | 24.93 |
| Sa | 308.67 | 18.74 |
| CaPhi | 293.92 | 24.27 |
| AzA | 285.98 | 23.23 |
| BABA | 285.94 | 26.34 |
| Sacch | 282.44 | 20.78 |
| MBS | 280.74 | 19.12 |
| KPhi | 276.78 | 24.26 |
| BTH | 271.03 | 23.15 |
| Ctrl. | 263.25 | 24.65 |
| Rib | 261.45 | 21.03 |
| CHT | 259.53 | 12.71 |
| Hx | 255.48 | 25.84 |
| Si | 254.51 | 24.27 |
| MeJA | 226.37 | 27.26 |

**Table S6.** Average time to reach to pupal stage (in days; n = 6).

| **Treatment** | **Days to pupa** | **Standard deviation** |
| --- | --- | --- |
| MeJA | 28.33 | 1.97 |
| BTH | 27.50 | 2.14 |
| Sacch | 26.83 | 2.11 |
| CHT | 26.50 | 1.50 |
| Si | 26.50 | 1.50 |
| Hx | 26.50 | 1.89 |
| Ctrl | 26.33 | 1.89 |
| KPhi | 25.50 | 1.61 |
| CaPhi | 25.50 | 1.89 |
| MBS | 25.50 | 1.89 |
| Rib | 25.33 | 1.80 |
| BABA | 24.83 | 1.95 |
| Thiam | 24.33 | 1.70 |
| AzA | 23.83 | 2.03 |
| SA | 23.83 | 2.03 |

**Table S7.** Statistical analysis of the physiological responses to drought stress of broccoli plants treated with salicylic acid (AS) shown in Figure 8. Data obtained from day one to day ten are presented. The different letters indicate the statistical significance determined by ANOVA (P < 0.001) with a post-hoc Tukey test (P < 0.05).
